# LncRNA LL22NC03-N14H11.1 promoted hepatocellular carcinoma progression through activating MAPK pathway to induce mitochondrial fission

**DOI:** 10.1038/s41419-020-2584-z

**Published:** 2020-10-07

**Authors:** Tingzhuang Yi, Hongcheng Luo, Fengxue Qin, Qi Jiang, Shougao He, Tonghua Wang, Jianwei Su, Sien Song, Xiaoshan Qin, Yueqiu Qin, Xihan Zhou, Zansong Huang

**Affiliations:** 1 Gastrointestinal Medicine, Affiliated Hospital of YouJiang Medical University For Nationalities, Baise, Guangxi 533000 P. R. China; 2 Laboratory Medicine, Affiliated Hospital of YouJiang Medical University For Nationalities, Baise, Guangxi 533000 P. R. China; 3 Blood transfusion Department, Affiliated Hospital of YouJiang Medical University For Nationalities, Baise, Guangxi 533000 P. R. China

**Keywords:** Cancer, Cancer genomics

## Abstract

Involvement of long non-coding RNAs (lncRNAs) in hepatocarcinogenesis has been largely documented. Mitochondrial dynamics is identified to impact survival and metastasis in tumors including hepatocellular carcinoma (HCC), but the underlying mechanism remains poorly understood. This study planned to explore the regulation of lncRNA LL22NC03-N14H11.1 on HCC progression and mitochondrial fission. Dysregulated lncRNAs in HCC are identified through circlncRNAnet and GEPIA bioinformatics tools. Biological function of LL22NC03-N14H11.1 in HCC was detected by CCK-8 assay, flow cytometry analysis, transwell invasion, and wound healing assays. Molecular interactions were determined by RNA immunoprecipitation, RNA pull-down, and co-immunoprecipitation assays. Results showed that LL22NC03-N14H11.1 was upregulated in HCC tissues and cells. Functionally, LL22NC03-N14H11.1 contributed to cell proliferation, migration, invasion, and epithelial-to-mesenchymal transition (EMT) in HCC. Moreover, LL22NC03-N14H11.1 facilitated mitochondrial fission in HCC cells. Mechanistically, LL22NC03-N14H11.1 recruited Myb proto-oncogene (c-Myb) to repress the transcription of leucine zipper-like transcription regulator 1 (LZTR1), so as to inhibit LZTR1-mediated ubiquitination of H-RAS (G12V), leading to the activation of mitogen-activated protein kinase (MAPK) signaling and induction of p-DRP1 (Serine 616). In conclusion, this study firstly revealed that lncRNA LL22NC03-N14H11.1 promoted HCC progression through activating H-RAS/MAPK pathway to induce mitochondrial fission, indicating LL22NC03-N14H11.1 as a novel potential biomarker for HCC treatment.

## Introduction

Hepatocellular carcinoma (HCC) is the third most fatal malignancy globally, taking up 90% of primary liver cancer cases^1^. Although during recent decades, surgery and interventional therapies have been greatly advanced, survival of HCC patients is still unsatisfactory^2^, which is mainly attributed to easy metastasis and frequent recurrence^3^. Therefore, further exploration of the precise mechanism behind HCC progression is urgently required.

Long non-coding RNAs (lncRNAs) are defined as a group of transcripts with a length over 200 nucleotides and lack of protein products^4,5^. Volumes of studies have offered convincing evidences that lncRNAs serve important roles in carcinogenesis of a wide range of cancer types^6,7^, including HCC^8,9^. Mechanistically, lncRNAs functioned through regulating target genes at epigenetic, transcriptional, and post-transcriptional levels^10,11^, and the mechanism varies depending on the cellular location of lncRNAs^12^. Here, we firstly discovered a novel lncRNA LL22NC03-N14H11.1 located at chr22:16,154,073–16,154,766 via bioinformatics analysis, and the results showed that LL22NC03-N14H11.1 was upregulated and had poor prognostic significance in HCC samples. Thus, we speculated that LL22NC03-N14H11.1 might participate in HCC progression and intended to probe into its role in HCC.

The morphology of mitochondria is highly dynamic and varies constantly through fission and fusion, helping cells adjust their morphology to satisfy the variable need of cells and adapt to the cellular environment^13^. Studies have shown that mitochondrial fission is increased in tumor cells, and that inhibiting mitochondrial fission can impede proliferation and induce apoptosis in several cancer types, such as colon cancer and lung cancer^14–16^. Also, facilitated mitochondrial fission can aggravate cancer metastasis through promoting migration and invasion^17,18^. Notably, Huang et al.^19^ stated that mitochondrial fission was increased in HCC and promoted HCC cell survival and autophagy. However, whether LL22NC03-N14H11.1 played a part in HCC via its regulation on mitochondrial fission has never been explored.

Moreover, highly conserved dynamin-related GTPases are recognized as primary regulators of mitochondrial dynamics, and the typical one of which is dynamin‐related protein 1 (DRP1)^13,20^. Phosphorylation of DRP1 at Serine 616 (S616) is proved to be required for the activation and recruitment of DRP1 to the mitochondrial membrane to induce mitochondrial fission^21^. For example, CDK1 phosphorylates Ser585 of rat DRP1, which is equivalent to human S616 during mitosis^22^. Protein disulfide isomerase (PDI1) facilitates DRP1 S616 phosphorylation and mitochondrial fission in CA1 neurons^23^. Multiple studies reveal that active ERK1/2 also contributes to the phosphorylation of DRP1 at S616^24^. Importantly, DRP1 S616 phosphorylation by the activation of RAS/MAPK (mitogen-activated protein kinase) signaling is demonstrated to be oncogenic in cancer cells. For example, RAS (G12V)-induced MAPK pathway led to DRP1 S616 phosphorylation and mitochondrial fission and tumor cell survival^25^. H-RAS (G12V) phosphorylated ERK1/2 and contributed to pancreatic cancer tumor growth through facilitating DRP1 S616 phosphorylation and mitochondrial fission^26^. To date, RAS/MAPK pathway has been largely reported to be involved in cell survival, apoptosis, and metastasis in cancers. However, the correlation of LL22NC03-N14H11.1 with RAS/MAPK signaling has never been investigated yet.

Leucine zipper-like transcription regulator 1 (LZTR1) is a Golgi protein belonging to BTB-Kelch superfamily^27^, which is generally known to function through interacting with Cullin3 (CUL3)-based E3 ubiquitin ligases^28,29^. LZTR1 has been reported to potentially participate in apoptosis and ubiquitination^27,30,31^. Several studies have revealed the tumor-suppressive role of LZTR1, and the germline and somatic mutations of LZTR1 in patients with glioblastoma and schwannomatosis^32–34^. Interestingly, it has been reported that LZTR1 is a conserved regulator of the ubiquitination of RAS family, leading to the degradation of both wild-type and mutant RAS members to inactivate MAPK pathway^35^. These findings indicated that LZTR1 might elicit its tumor-suppressive function through MAPK pathway, and that LZTR1 might be related to mitochondrial fission. However, the function and mechanism of LZTR1 in HCC and its association with mitochondrial fission have never been revealed.

Therefore, the present study aimed to uncover the role and mechanism of LL22NC03-N14H11.1 in HCC and its potential regulation on mitochondrial fission.

## Materials and methods

### Clinical specimen collection

Sixty-two patients diagnosed with HCC were recruited for collecting tumor and adjacent non-tumor tissue samples between 2013 and 2018, with the approval of Ethics Committee of Affiliated Hospital of YouJiang Medical University For Nationalities. Paired non-tumor tissues were at least 5 cm away from the tumor margin. Samples were frozen immediately in liquid nitrogen after surgical resection and stored at −80 °C until used for total RNA extraction. Patients treated with radiotherapy or chemotherapy before surgery were excluded, and all subjects had provided the written informed consent.

### Cell culture

Normal liver cell line THLE-3, human renal epithelial cell line 293T, and HCC cell lines (Hep3B, SNU-449, LM3, Huh7 and SK-HEP-1) were acquired from the American Type Culture Collection (Manassas, VA, USA). In accordance with the protocol of Dulbecco’s modified Eagle’s medium (DMEM) (Gibco, Rockville, MD, USA) containing 1% penicillin/streptomycin (Gibco) and 10% fetal bovine serum (FBS; Gibco), cells were grown routinely in 5% CO_2_ at 37 °C. The medium was changed every 3 days, and cell passage was performed when the cells grew up to 80–90% confluence.

### Reagent and antibodies

The commercially available mitochondrial division inhibitor, Mdivi-1, was from Sigma-Aldrich (Saint-Louis, MO, USA) and utilized for treating SK-HEP-1 and Huh7 cells, with DMSO (Sigma-Aldrich) as the control group. Translation inhibitor cycloheximide chase was also from Sigma-Aldrich for assessing protein stability. The primary antibodies against cleaved (c)-caspase-3 (ab2302, Abcam), total (t)-caspase-3 (ab13847, Abcam), c-caspase-6 (ab2326, Abcam), t-caspase-6 (ab185645, Abcam), Bax (ab32503, Abcam), Bcl-2 (ab32124, Abcam), E-cadherin (ab40772, Abcam), N-cadherin (ab76057, Abcam), MMP2 (ab37150, Abcam), MMP7 (ab5706, Abcam), DRP1 (ab184247, Abcam), PDI1 (ab4644, Abcam), CDK1 (ab18, Abcam), H-RAS (G12V) (ab140571, Abcam), LZTR1 (ab106655, Abcam), c-Myb (ab109127, Abcam), and GAPDH (ab245356, Abcam), as well as secondary antibodies were all obtained from Abcam (Cambridge, MA, USA). Antibodies against p-DRP1 (S616) (#3455S, Cell Signaling Technology, Danvers, MA, USA), p-ERK1/2 (#4370S, Cell Signaling Technology), and ERK1/2 (#4695S, Cell Signaling Technology) were obtained from Cell Signaling Technology (Danvers, MA, USA).

### Quantitative real-time PCR

Total cellular RNA preparation and complementary DNA (cDNA) synthesis were separately conducted via TRIzol (Invitrogen, Carlsbad, CA, USA) and High-Capacity cDNA Reverse Transcription Kit (Thermo Fisher Scientific, Waltham, MA, USA). SYBR Green PCR Master Mix (Takara, Kyoto, Japan) was used for quantitative real-time PCR (RT-qPCR) analysis on ABI Prism 7900HT sequence detector (Applied Biosystems, Foster City, CA, USA). Calculation of relative gene expression was achieved using the 2^−ΔΔCT^ method. GAPDH and U6 acted as endogenous controls to normalize the data. The specific PCR primers were presented as follows: LL22NC03-N14H11.1, forward primer, 5′-GAGTCTGGGGATCAGCATCG, reverse primer, 5′-TCCAGGGGGCTGGATAATGA-3′; LINC00152, forward primer, 5′-TACTGCTGAGAGACCCCCTC-3′, reverse primer, 5′-TAGCCAAAGGTTGGAAGCCA-3′; RP11-620J15.3, forward primer, 5′-TGCGGGACACGTTATCACAA-3′, reverse primer, 5′-TTCAGCTCTCACGTTCCACC-3′; H-RAS, forward primer, 5′-ACCTGTTCTGGAGGACGGTA-3′, reverse primer, 5′-TCTCAACCACGCACCCAAAT-3′; LZTR1, forward primer, 5′-AGCGTGGACTTCGACCATAG-3′, reverse primer, 5′-GCCAGCGATGCACTGTTTC-3′; C-Myb, forward primer, 5′-GCGAAGGAGTTCTAAGGCGA-3′, reverse primer, 5′-CCGGTAGCTGTCCTGTGAAG-3′; U1, forward primer, 5′-ACGGACGAAACAATGACCGT-3′, reverse primer, 5′-TTATTCACGCGTACTCCGCA-3′; GAPDH, forward primer, 5′-CTGGGCTACACTGAGCACC-3′, reverse primer, 5′-AAGTGGTCGTTGAGGGCAATG-3′; U6, forward primer, 5′-GCAGACCGTTCGTCAACCTA-3′, reverse primer, 5′-AATTCTGTTTGCGGTGCGTC-3′.

### Subcellular fractionation

Nuclear and cytoplasmic fractions of SK-HEP-1 or Huh7 cells were separated utilizing the PARIS Kit (Thermo Fisher). Cell samples were first lysed in cell fractionation buffer. The pellet washed with TSE buffer (10 mM Tris, 300 mMsucrose, 1 mM EDTA, 0.1% NP40 PH 7.5) at 4000 g for 5 min in a tabletop centrifuge at 4 ℃. The resulting supernatant discarded and the pellets were nucleus. The resulting supernatant from the first round of differential centrifugation was sedimented for 150 min at 14000 rpm in a tabletop centrifuge. The resulting pellets were membranes and the supernatant were cytoplasm. RT-qPCR was followed to determine the location of LL22NC03-N14H11.1, with GAPDH or U6 as the cytoplasmic or nuclear control. The experiment was conducted in triplicate.

### Fluorescence in situ hybridization

The RNA fluorescence in situ hybridization (FISH) probe for LL22NC03-N14H11.1 was designed and produced at RiboBio (Guangzhou, China). Cell samples were fixed in 4% formaldehyde for 15 min before washing in phosphate-buffered saline (PBS), and then cultured with pepsin and dehydrated. The air-dried cells were incubated with FISH probe in the hybridization buffer. Nuclei were counterstained with 4′,6-diamidino-2-phenylindole (DAPI) and cells were observed using laser scanning confocal microscope (Zeiss, Jena, Germany). The experiment was conducted in triplicate.

### Transfection

Specific short hairpin RNAs (shRNAs) against LL22NC03-N14H11.1 (sh-LL22NC03-N14H11.1#1/2/3), LZTR1 (sh-LZTR1), ELF1 (sh-ELF1), AR (sh-AR) or c-Myb (sh-c-Myb) and corresponding negative controls (sh-NC) were acquired from GeneChem (Shanghai, China). The interference sequences were presented as follows: sh-NC, 5′-CCGGGGATTCCTATCCCCTGTCAATCTCGAGTTTGACAGGGGAAACCAATCGTTTTTG-3′; sh-LL22NC03-N14H11.1#1, 5′-CCGGGCACTGGTATGGGCTGTCTATCTCGAGATAGACAGCCCATACCAGTGCTTTTTG-3′; sh-LL22NC03-N14H11.1#2, 5′-CCGGGGAATCAGGCCTCCCAAATTTCTCGAGAAATTTGGGAGGCCTGATTCCTTTTTG-3′; sh-LL22NC03-N14H11.1#3, 5′-CCGGGATGGAGAAAGCACTCACCTACTCGAGTAGGTGAGTGCTTTCTCCATCTTTTTG-3′; sh-NC, 5′-CCGGGCTTAAGTTCAGAAGACTTGGACTCGAGCGAATTCAAGTCTTCTGAACCTTTTTTG-3′, sh-LZTR1, 5′-CCGGTCAGGGTCTCGGGCTACAGGGCCTCGAGAGTCCCAGAGCCCGATGTCCCGTTTTTG-3′; sh-NC, 5′-CCGGTAGCTAGATAAAGTTATAGAATCTCGAGATCGATCTATTTCAATATCTTATTTTTG-3′, sh-ELF1, 5′-CCGGTTCCTCCGTCTCCGTGAATACGCTCGAGAAGGAGGCAGAGGCACTTATGCTTTTTG-3′; sh-NC, 5′-CCGGAATTGTATAGAAACTATATCTGCTCGAGTTAACATATCTTTGATATAGACTTTTTG-3′, sh-AR, 5′-CCGGAAGTTCATAATTCTCTGTCTGACTCGAGTTCAAGTATTAAGAGACAGACTTTTTTG-3′; sh-NC, 5′-CCGGACTTGAAAGCGCAAGGAATTAGCTCGAGTGAACTTTCGCGTTCCTTAATCTTTTTG-3′, sh-c-Myb, 5′-CCGGCCGCTCCCTAGGCGTGCGATTACTCGAGGGCGAGGGATCCGCACGCTAATTTTTTG-3′. The pcDNA3.1 vector targeting DRP1 or c-Myb, as well as the active H-Ras (G12V) expressed from retroviral vector LZRS, was applied for gene overexpression. These plasmids were transfected into SK-HEP-1 or Huh7 cells for 48 h in 6-well plates (1 × 10^6^ cells/well), by Lipofectamine 3000 (Invitrogen, Carlsbad, CA, USA). The experiment was conducted in triplicate.

### CCK-8

Transfected SK-HEP-1 or Huh7 cells were prepared in 96-well plates containing complete medium with 5 × 10^3^ cells in each well, and 10 µL of CCK-8 solution (Dojindo, Kumamoto, Japan) was added into each well for 2 h to detect cell viability. The optical density value at 450 nm was recorded at 0, 24, 48, 72, and 96 h using a microplate reader (Dynex Technologies, West Sussex, UK). The experiment was conducted in triplicate.

### Colony formation

Transfected SK-HEP-1 or Huh7 cells at the logarithmic growth phase were trypsinized, harvested, and planted into 6-well plates for 2 weeks, at the density of 500 cells per well. After washing in PBS, colonies containing more than 50 cells were fixed in methanol (Sigma-Aldrich), dyed in crystal violet (Sigma-Aldrich), and eventually counted manually. The experiment was conducted in triplicate.

### Flow cytometry of apoptosis

Annexin‐V‐FITC/Propidium Iodide (PI) Apoptosis Detection Kit (BD Biosciences, Franklin Lakes, NJ, USA) was obtained for flow cytometry analysis. A total of 1 × 10^6^ cells of SK-HEP-1 or Huh7 were collected after transfection, and plated into 6-well plates for treatment in 100 μL of 1× Binding Buffer containing 5 μL of PI and 5 μL of Annexin‐V‐FITC. After culturing in a dark room for 15 min, cell apoptosis of transfected SK-HEP-1 or Huh7 cells was analyzed by FACScan (BD Biosciences) and FlowJo V10 software (Tree Star, Ashland, OR, USA). The experiment was conducted in triplicate.

### Western blotting

Total cellular protein samples were prepared using RIPA lysis buffer and subjected to sodium dodecyl sulfate (SDS)-polyacrylamide gel, following moving onto PVDF membranes (Bio-Rad Laboratories, Hercules, CA, USA). After blocking with 5% nonfat milk, membranes were cultivated with primary antibodies at 4 °C all night. Following washing in Tris‑buffered saline/Tween-20 (TBST), membranes were probed with secondary antibodies at room temperature for 2 h. The band density was analyzed with application of the ECL luminous liquid (Pierce, Rockford, IL, USA) and quantified via the ImageJ software (National Institutes of Health, USA). The experiment was conducted in triplicate.

### Invasion assay

Cell invasion was explored by 24-well Transwell chambers (8-mm pore size; Corning Incorporated, Big Flats, NY, USA) pre-coated with Matrigel (BD Biosciences). The upper chamber was added with 2 × 10^4^ transfected cells in serum-free medium, while lower chamber was filled with complete culture medium containing 10% FBS. Invaded cells on the bottom of membrane were fixed by 4% formaldehyde after 24 h, stained with crystal violet, and counted under the optical microscope (Thermo Fisher). The experiment was conducted in triplicate.

### Wound healing

Transfected SK-HEP-1 or Huh7 cells (1 × 10^6^) were seeded into 6-well plates and cultured until cells reached 80–90% confluence. The following day, cells were treated with a 200 μL filter tip for creating the wound area, followed by 24 h of incubation at 37 °C in serum-free medium. The distance of wound healing was photographed under a microscope (Olympus, Tokyo, Japan) at 0 or 24 h. The experiment was conducted in triplicate.

### Immunofluorescence

Transfected SK-HEP-1 or Huh7 cells in PBS (Sigma-Aldrich) were plated onto culture slides for 24 h, incubated in 1% paraformaldehyde (Sigma-Aldrich) for 10 min, permeabilized in methanol, and blocked with 0.8% bovine serum albumin (Sigma-Aldrich) for 10 min. After incubation all night with primary antibodies against E-cadherin and N-cadherin, secondary antibodies were added for 2 h. Coverslips were mounted on glass slides after staining with DAPI (Sigma-Aldrich) for 10 min, and were finally examined using the TE2000-U microscope (Nikon, Tokyo, Japan). The experiment was conducted in triplicate.

### Mitochondrial staining and mitochondrial fission analysis

SK-HEP-1 or Huh7 cells were transplanted onto coverslips and transfected. Cells were centrifuged for 5 min at room temperature for treatment with 0.1 μM of MitoTracker Red CMXRos (Molecular Probes, Thermo Fisher) for 30 min. After washing, images were taken using laser scanning confocal microscope. The experiment was conducted in triplicate.

### Co-immunoprecipitation

Transfected cells were reaped from immunoprecipitation lysis buffer, centrifuged, and then cell lysates were incubated overnight with indicated antibodies at a constant speed at 4 °C. Normal immunoglobulin G (IgG) was seen as a negative control. Following culturing with protein A-sepharose beads, the antigen–antibody mixture was washed three times with IP lysis buffer, eluted, and subjected to immunoblotting (Western blot). The experiment was conducted in triplicate.

### Ubiquitination assay

Transfected cells were incubated in hot lysis buffer containing 1% SDS and 10 mM of *N*-ethylmaleimide (Sigma-Aldrich) and boiled at 100 °C for 10 min. Cell lysates were diluted in SDS-free cell lysis buffer and mixed with anti-Flag-M2 agarose and assessed by western blotting. The experiment was conducted in triplicate.

### Luciferase reporter assay

The wild-type or mutant sequences of c-Myb in LZTR1 promoter were sub-cloned into pGL3-basic vector (Promega, Madison, WI, USA) and co-transfected into 293T cells with pcDNA3.1/c-Myb or pcDNA3.1. The pGL3-LZTR1 promoter vector was co-transfected into SK-HEP-1 or Huh7 cells with sh-LL22NC03-N14H11.1#1/2 or sh-NC. Luciferase activities were studied with Dual-Luciferase reporter assay system (Promega), using *Renilla* luciferase as the internal control. The experiment was conducted in triplicate.

### RNA pull-down assay

RNA pull-down assay was studied by Pierce Magnetic RNA-Protein Pull-Down Kit (Thermo Fisher Scientific, Waltham, MA, USA). Cell protein lysates of SK-HEP-1 or Huh7 were acquired by using RIPA lysis buffer, and then incubated with biotinylated RNA probes (LL22NC03-N14H11.1 biotin or LL22NC03-N14H11.1 no biotin). The streptavidin-coated magnetic beads (Invitrogen) were added to capture the RNA–protein mixture. Western blot was applied for analysis of the enrichment of proteins pulled down in indicated groups. The experiment was conducted in triplicate.

### RNA immunoprecipitation

Magna RIP™ RNA-Binding Protein Immunoprecipitation Kit was bought from Millipore (California, USA). Cell lysates were prepared using RNA immunoprecipitation (RIP) lysis buffer, and then cultivated with RIP buffer and magnetic beads conjugated to anti-c-Myb antibody, with anti-IgG antibody as a negative control. Besides, the interaction between U1 and SNRNP70 was used as a positive control for such assays. After adding proteinase K, precipitates were assayed by RT-qPCR. The experiment was conducted in triplicate.

### Chromatin immunoprecipitation

Chromatin immunoprecipitation (ChIP) assay was undertaken with the application of EZ ChIP™ Chromatin Immunoprecipitation Kit (Millipore), following the standard method. Cells were treated in formaldehyde for 10 min for generating the DNA–protein cross-links. Then, cell lysates were sonicated to acquire chromatin fragments of 200–300 bp and immunoprecipitated with antibodies against c-Myb and control IgG. RT-qPCR was followed for the retrieved precipitated chromatin DNA. The experiment was conducted in triplicate.

### Tumor xenograft model

Four-week-old male BALB/c nude mice were purchased from Shi Laike Company (Shanghi, China) and maintained in SPF-grade, pathogen-free animal lab. Tumor xenograft assay was performed via injecting nude mice subcutaneously with 1 × 10^6^ SK-HEP-1 cells transfected with sh-LL22NC03-N14H11.1#1, sh-LL22NC03-N14H11.1#1 + sh-LZTR1, or sh-NC (mice were randomly divided into three groups). Tumor volume was recorded every 4 days. Four weeks later, mice were killed by cervical dislocation and tumors were weighted. Animal studies were approved by the Animal Ethics Committee of Affiliated Hospital of YouJiang Medical University For Nationalities.

### TUNEL assay

To perform terminal deoxynucleotidyl transferase dUTP nick-end labeling (TUNEL) assay in xenograft tissues, One-Step TUNEL Apoptosis Assay Kit was commercially acquired and used as instructed (Beyotime, Shanghai, China). After treatment with proteinase K for 15 min at room temperature, sections were fixed in 4% paraformaldehyde for 1 h and permeabilized with 0.1% Triton-X100 for 2 min, followed by incubation with TUNEL Assay Kit. The fluorescence microscope (Leica, Heerbrugg, Canton of St. Gallen, Switzerland) was utilized for observing the TUNEL-stained and DAPI-stained sections. The apoptotic nuclei were determined as the TUNEL and DAPI-positive nuclei located within tumor tissues. The experiment was conducted in triplicate.

### Immunohistochemistry

The tumor tissues were acquired from xenograft assay and then fixed in 4% paraformaldehyde, dehydrated, and embedded in paraffin. Four-millimeter-thick sections were cut from paraffin-embedded xenograft tissues and deparaffinized, followed by cultivation with antibodies against Ki67 (Abcam), proliferating cell nuclear antigen (PCNA) (Abcam), E-cadherin and N-cadherin overnight at 4 °C, and with biotinylated second antibody for 30 min at 37 °C. The experiment was conducted in triplicate.

### Hematoxylin and eosin staining

Tissues from xenograft model were immobilized for 24 h utilizing 4% formaldehyde and paraffin-embedded, followed by sectioned and stained with hematoxylin and eosin (HE) (Sigma-Aldrich). The optical microscope (Nikon) was applied for analyzing. The experiment was conducted in triplicate.

### Statistical analysis

Data (in line with normal distribution) from assays conducted in triplicate were listed as mean ± SD. Statistical analysis was conducted by SPSS V.19.0 (SPSS, Chicago, IL, USA) or Prism 6 (GraphPad Software, San Diego, CA, USA). The overall survival was plotted via the Kaplan–Meier method and compared by log-rank test. Gene expression correlation was analyzed with Pearson’s method. Student’s *t* test or analysis of variance was appropriately adopted for significant differences in groups, with *P* < 0.05 as threshold.

## Results

### LL22NC03-N14H11.1 was upregulated in HCC and mainly located in the nucleus

**Fig. 1:**
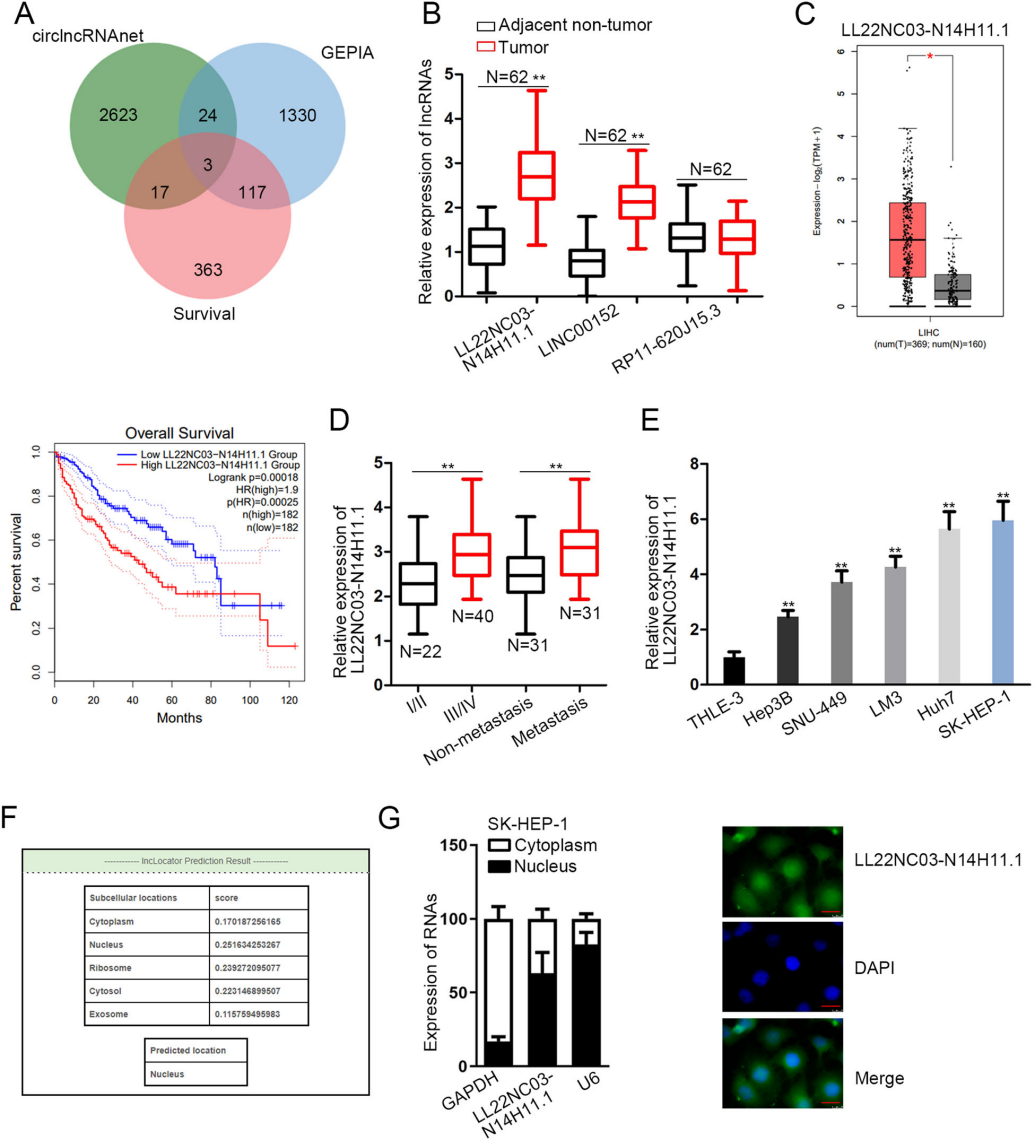
Expression and cellular localization of LL22NC03-N14H11.1 in HCC. **a** Differentially expressed lncRNAs in HCC samples from circlncRNAnet data (*P* < 0.05, Log 2 FC > 1) were compared to the data of differentially expressed genes and genes associated with survival in HCC samples from GEPIA2. Intersection of Venn pattern showed that three lncRNAs, LL22NC03-N14H11.1, LINC00152, and RP11-620J15.3 were sorted out. **b** RT-qPCR data of the levels of three above-mentioned lncRNAs in 62 pairs of HCC tissues and paired non-tumorous ones. **c** GEPIA data of LL22NC03-N14H11.1 upregulation in LIHC samples and the association of high LL22NC03-N14H11.1 level with low survival in LIHC patients. **d** RT-qPCR data of LL22NC03-N14H11.1 level in HCC tissues at stage I/II (*N* = 22) and stage III/IV (*N* = 40), and in non-metastasis HCC tissues (*N* = 31) and metastasis HCC tissues (*N* = 31). **e** RT-qPCR results of LL22NC03-N14H11.1 level in five HCC cell lines and normal cell line. **f** Prediction results of lncLocator indicated LL22NC03-N14H11.1 localization mainly in the nucleus. **g** Subcellular fractionation and FISH staining confirmed that LL22NC03-N14H11.1 was mainly localized in the nucleus in SK-HEP-1 cells. Scale bar: 25 μm. *P < 0.05; **P < 0.01.

First, to identify the dysregulated lncRNAs in HCC, we browsed circlncRNAnet (http://120.126.1.61/circlnc/circlncRNAnet/lncRNA_TCGA/index.php) and identified 2667 upregulated lncRNAs in HCC samples (*P* < 0.05, log 2 fold change (FC) > 1). Then, we analyzed these lncRNAs in the GEPIA database (Gene Expression Profiling Interactive Analysis) (http://gepia2.cancer-pku.cn/#index), finding that three lncRNAs, LL22NC03-N14H11.1, LINC00152, and RP11-620J15.3, presented significant upregulation in liver HCC (LIHC) samples and had potential prognostic values in LIHC patients (Fig. 1a). Therefore, we examined the expression of the three lncRNAs in HCC tissues collected from our institute. RT-qPCR data revealed that both LL22NC03-N14H11.1 and LINC00152 were highly expressed in 62 HCC samples vs. the matched adjacent non-tumor samples, and the upregulation of LL22NC03-N14H11.1 was more significant than LINC00152 (Fig. 1b). Since LINC00152 has already been reported as an oncogene in HCC by several previous studies^36–38^, we focused on the role of LL22NC03-N14H11.1 in HCC here. The significant elevation of LL22NC03-N14H11.1 in LIHC samples (*n* = 369) vs. normal tissues (*n* = 160) and the positive association of high LL22NC03-N14H11.1 level with low overall survival in LIHC patients (*p* = 0.00018) according to the GEPIA database were shown in Fig. 1c. Besides, we verified that LL22NC03-N14H11.1 was highly expressed in HCC patients at advanced clinical stages (stage III/IV, *n* = 40) compared with primary stages (stage I/II, *n* = 22) (Fig. 1d). Also, LL22NC03-N14H11.1 level was higher in metastatic HCC patients (*n* = 31) than non-metastatic HCC patients (*n* = 31) (Fig. 1d). These data indicated that LL22NC03-N14H11.1 potentially participated in tumor growth and metastasis in HCC. Moreover, upregulation of LL22NC03-N14H11.1 was validated in five HCC cell lines (Hep3B, SNU-449, LM3, Huh7, and SK-HEP-1) vs. the normal THLE-3 cells, and SK-HEP-1 and Huh7 cells presented the highest LL22NC03-N14H11.1 level (Fig. 1e). In addition, through lncLocator (http://www.csbio.sjtu.edu.cn/bioinf/lncLocator/), we found that LL22NC03-N14H11.1 was mainly expressed in the nucleus (score = 0.252) (Fig. 1f). Subcellular fractionation and FISH staining also confirmed that LL22NC03-N14H11.1 was expressed in the nucleus more than in the cytoplasm of SK-HEP-1 cells (Fig. 1g). In collection, LL22NC03-N14H11.1 was upregulated in HCC and was mainly expressed in the nucleus of HCC cells.

### LL22NC03-N14H11.1 aggravated proliferation, prevented apoptosis, drove migration, invasion, and EMT in HCC in vitro

**Fig. 2:**
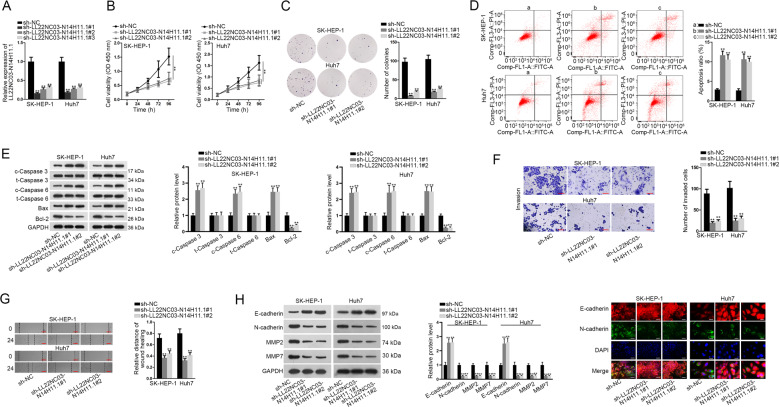
Biological function of LL22NC03-N14H11.1 silence in HCC cells. **a** RT-qPCR data of LL22NC03-N14H11.1 level in SK-HEP-1 and Huh7 cells transfected with sh-NC or sh-LL22NC03-N14H11.1#1/2/3. **b** Cell viability of SK-HEP-1 and Huh7 cells at indicated time was evaluated by CCK-8 under indicated transfection. **c** Representative images and quantification of colonies formed by two HCC cells under indicated transfection. **d** Flow cytometry analysis of apoptotic HCC cells under LL22NC03-N14H11.1 silence vs. control. **e** Western blots of cleaved and total caspase-3 and caspase-6, Bax, and Bcl-2 in 2 HCC cells. **f** Representative pictures of invading HCC cells and quantification under LL22NC03-N14H11.1 depletion were obtained via transwell assay. Scale bar: 100 μm. **g** Images of wound width of HCC cells of each group and quantification of relative distance of wound healing obtained from wound healing assay. **h** Western blots of E-cadherin, N-cadherin, MMP2, and MMP7 in HCC cells with LL22NC03-N14H11.1 silence. **i** IF staining of E-cadherin and N-cadherin in HCC cells of each group. Scale bar: 50 μm. **P < 0.01.

Next, we detected the biological role of LL22NC03-N14H11.1 in HCC through in vitro loss-of-function assays conducted in Huh7 and SK-HEP-1 cells expressing high LL22NC03-N14H11.1 level. Successfully, RT-qPCR data confirmed the overt silence of LL22NC03-N14H11.1 by three shRNAs in both the HCC cell lines, and sh-LL22NC03-N14H11.1#1/2 showed higher knockdown efficiency (Fig. 2a). Therefore, cells transfected with sh-LL22NC03-N14H11.1#1/2 were applied for subsequent experiments. Consequently, silence of LL22NC03-N14H11.1 attenuated the viability and colony formation ability of two HCC cell lines (Fig. 2b, c). According to flow cytometry analysis, ratio of apoptotic HCC cells increased under the silence of LL22NC03-N14H11.1 (Fig. 2d). Concordantly, levels of pro-apoptotic genes (*cleaved-caspase-3*, *cleaved-caspase-6*, and *Bax*) increased, whereas the level of anti-apoptotic gene (*Bcl-2*) decreased responding to LL22NC03-N14H11.1 silence in HCC cells, with total caspase-3 and caspase-6 unchanged (Fig. 2e), further suggesting that LL22NC03-N14H11.1 depletion increased apoptosis in HCC cells. Besides, the effect of LL22NC03-N14H11.1 on metastasis of HCC cells was assessed. We observed the retarded invasion of HCC cells under LL22NC03-N14H11.1 silence through a transwell system (Fig. 2f). Scratch wound assay depicted that LL22NC03-N14H11.1 knockdown inhibited migration of HCC cells (Fig. 2g). Additionally, EMT markers were examined in HCC cells. Western blot demonstrated that under the knockdown of LL22NC03-N14H11.1, E-cadherin level increased, whereas N-cadherin, MMP2, and MMP7 levels decreased in HCC cells (Fig. 2h). Meanwhile, IF analysis indicated the augmented E-cadherin level and declined N-cadherin staining in SK-HEP-1 and Huh7 cells in response to LL22NC03-N14H11.1 inhibition (Fig. 2i). These data suggested that LL22NC03-N14H11.1 silence hindered EMT progression in HCC cells. Meanwhile, we also implemented gain-of-function assays in Hep3B and SNU-499 cells. As expected, after elevating LL22NC03-N14H11.1 expression in these two cells, the viability and proliferative ability were greatly strengthened (Supplementary Fig. S1A–C). Also, enhanced level of LL22NC03-N14H11.1 led to promoted motility in Hep3B and SNU-499 cells (Supplementary Fig. S1D, E). Besides, we observed facilitated EMT process in LL22NC03-N14H11.1-upregulated HCC cells (Supplementary Fig. S1F–G). Together, these data indicated that LL22NC03-N14H11.1 aggravated proliferation, migration, and invasion, as well as EMT process in HCC in vitro.

### LL22NC03-N14H11.1 promoted HCC progression through inducing p-DRP1 (S616) and facilitating mitochondrial fission

**Fig. 3:**
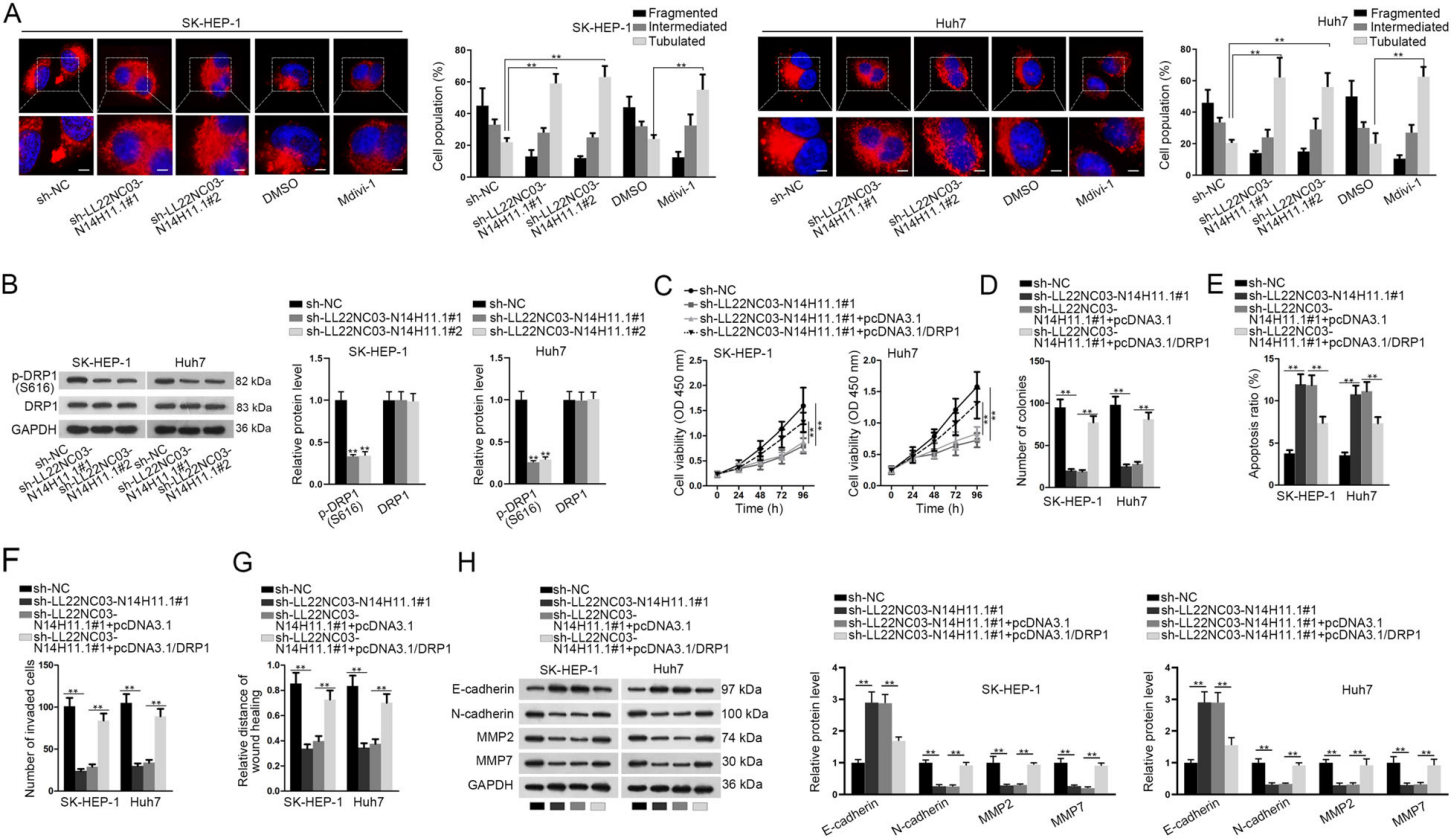
LL22NC03-N14H11.1 positively regulated mitochondrial fission through DRP1 S616 phosphorylation. **a** Mitochondrial fission was monitored by MitoTracker Red staining and quantified. Scale bar: 3 μm. **b** Western blots of the levels of p-DRP1 (S616) and total DRP1 in HCC cells with LL22NC03-N14H11.1 silence. **c–g** SK-HEP-1 and Huh7 cells were transfected with sh-NC, sh-LL22NC03-N14H11.1#1, sh-LL22NC03-N14H11.1#1 + pcDNA3.1, or sh-LL22NC03-N14H11.1#1 + pcDNA3.1/DRP1. Proliferation, apoptosis, invasion, and migration were evaluated by CCK-8, colony formation, flow cytometry, transwell invasion, and wound healing assays, respectively. **h** Western blots of E-cadherin, N-cadherin, MMP2, and MMP7 in HCC cells of each group. **P < 0.01.

A recent study reported that facilitated mitochondrial fission improved the survival and impeded apoptosis in HCC cells^19^. Moreover, studies have shown that inactivation of mitochondrial fission constrained cell migration and invasion in cancer^17,18^. Therefore, we wondered whether LL22NC03-N14H11.1 could regulate mitochondrial fission in HCC cells. MitoTracker Red staining analysis demonstrated that the mitochondrial elements were elongated and interconnected under LL22NC03-N14H11.1 knockdown or the treatment of Midiv-1, the selective inhibitor of DRP1, which is known to be the key regulator of mitochondrial fission (Fig. 3a). However, opposite phenomenon was observed in Hep3B and SNU-499 cells under enhanced expression of LL22NC03-N14H11.1 (Supplementary Fig. S2A). In addition, western blot analysis presented that LL22NC03-N14H11.1 knockdown reduced, whereas its upregulation boosted S616 phosphorylation of DRP1, while the total protein level of DRP1 was not changed under the above conditions (Fig. 3b, Supplementary Fig. S2B). Previous studies proved that p-DRP1 (S616) was required for the facilitation of mitochondrial fission and also for tumor growth^23,26^. Hence, these findings suggested that LL22NC03-N14H11.1 positively regulated mitochondrial fission in HCC cells through inducing p-DRP1 (S616). Later, we tried to examine whether LL22NC03-N14H11.1 influenced HCC progression through mitochondrial fission. As anticipated, it was proved that the hindered mitochondrial fission in LL22NC03-N14H11.1-silenced cells was normalized due to recovered p-DRP1 level upon DRP1 overexpression (Supplementary Fig. S2C, D). Thereafter, we confirmed that overexpression of DRP1 rescued the proliferation of HCC cells inhibited by sh-LL22NC03-N14H11.1#1 (Fig. 3c, d). Inductive effect of sh-LL22NC03-N14H11.1#1 on HCC cell apoptosis was abrogated by DRP1 overexpression (Fig. 3e). Invasion and migration of HCC cells restrained by sh-LL22NC03-N14H11.1#1 were restored by co-transfection of pcDNA3.1/DRP1 (Fig. 3f–g). The increase of E-cadherin and decrease of N-cadherin, MMP7, and MMP2 under LL22NC03-N14H11.1 knockdown were reversed by the overexpression of DRP1 (Fig. 3h). Jointly, it was indicated that LL22NC03-N14H11.1 promoted HCC progression through inducing p-DRP1 (S616)-facilitated mitochondrial fission.

### LL22NC03-N14H11.1 inhibited LZTR1-mediated ubiquitination of H-RAS (G12V) and activated MAPK pathway to induce p-DRP1 (S616)

**Fig. 4:**
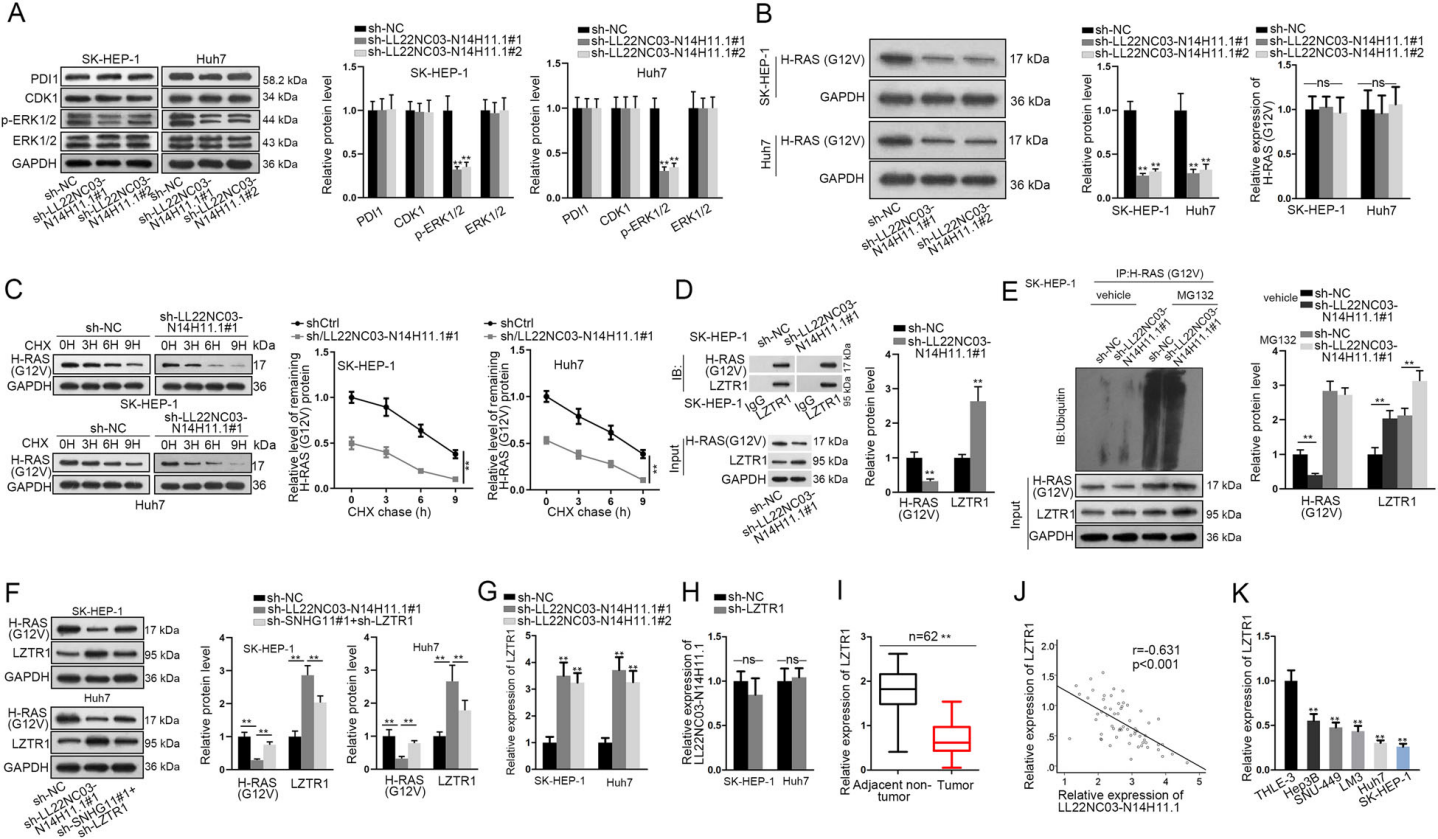
LL22NC03-N14H11.1 inhibited H-RAS (G12V) ubiquitination by inhibiting LZTR1. **a** Western blot results of PDI1, CDK1, p-ERK1/2, and total ERK1/2 in HCC cells under LL22NC03-N14H11.1 silence. **b** Western blot and RT-qPCR detected the expression of H-RAS (G12V) in Huh7 and SK-HEP-1 cells under LL22NC03-N14H11.1 silence. **c** Cycloheximide (CHX) was added to inhibit protein generation. Protein level of H-RAS (G12V) in SK-HEP-1 and Huh7 cells under LL22NC03-N14H11.1 silence was detected by western blot and quantified at 0, 3, 6, and 9 h after CHX treatment. **d** CoIP followed by western blot analyzed the levels of H-RAS (G12V) and LZTR1 in the binding complex of LZTR1 and control IgG, as well as their levels in input. **e** CoIP assay determined the ubiquitination level of H-RAS (G12V) in SK-HEP-1 cells under LL22NC03-N14H11.1 silence with or without MG132 treatment, and the level of input H-RAS (G12V) and LZTR1 was also detected via western blot. **f** Western blots of H-RAS (G12V) and LZTR1 in HCC cells under LL22NC03-N14H11.1 silence. **g** RT-qPCR analysis of LZTR1 level in HCC cells under LL22NC03-N14H11.1 silence. **h** RT-qPCR analysis of LL22NC03-N14H11.1 level in HCC cells under LZTR1 silence. **i** RT-qPCR analysis of LZTR1 level in 62 pairs of HCC tissues and matched non-tumorous tissues. **j** Negative correlation between LZTR1 and LL22NC03-N14H11.1 in HCC tissues was confirmed by Pearson’s correlation curve. **k** RT-qPCR analysis of LZTR1 in HCC cell lines vs. normal cell line. **P < 0.01; n.s. no significance.

Thereafter, we interrogated how LL22NC03-N14H11.1 induced p-DRP (S616). Several genes that were identified to regulate p-DRP (S616) were detected, including PDI1^23^, CDK1^39^, and ERK1/2^26^. Western blot data illustrated that knockdown of LL22NC03-N14H11.1 resulted in no significant change in the levels of PDI1 or CDK1, but led to decreased p-ERK1/2 level, while total ERK1/2 unchanged (Fig. 4a). ERK1/2 are known to be key regulators in MAPK pathway, and it has been reported that H-RAS (G12V) activated MAPK pathway so that ERK1/2 contributed to p-DRP (S616) and mitochondrial fission, which then drove tumor growth in pancreatic cancer^26^. Therefore, we detected the regulation of LL22NC03-N14H11.1 on active H-RAS (G12V) in two HCC cell lines. As a result, knockdown of LL22NC03-N14H11.1 reduced the level of H-RAS (G12V) at the protein level rather than the messenger RNA (mRNA) level in HCC cells (Fig. 4b). Furthermore, we found that knockdown of LL22NC03-N14H11.1 impaired the stability of H-RAS (G12V) proteins in HCC cells (Fig. 4c).

Former studies stated that LZTR1 was a regulator of ubiquitination of RAS family, and both mutant and wild-type of RAS could be ubiquitinated and degraded by LZTR1^35^. We tried to examine whether LL22NC03-N14H11.1 influenced the regulation of LZTR1 on H-RAS (G12V). CoIP assays showed that in SK-HEP-1 cells, H-RAS (G12V) was enriched in the precipitates of anti-LZTR1, and knockdown of LL22NC03-N14H11.1 enhanced such enrichment, with the input H-RAS (G12V) level decreased and LZTR1 level increased (Fig. 4d). Also, the ubiquitination of H-RAS (G12V) was induced under the knockdown of LL22NC03-N14H11.1, with the input H-RAS (G12V) level decreased and LZTR1 level increased (Fig. 4e). These results indicated that LL22NC03-N14H11.1 reduced the interaction between LZTR1 and H-RAS (G12V) by reducing LZTR1 expression. We confirmed through western blot that knockdown of LL22NC03-N14H11.1 decreased H-RAS (G12V) level and increased LZTR1 level, and such results could be reversed by the knockdown of LZTR1 in HCC cells (Fig. 4f). Additionally, silence of LL22NC03-N14H11.1A induced the mRNA level of LZTR1, but knockdown of LZTR1 had no impact on the level of LL22NC03-N14H11.1 in HCC cells (Fig. 4g, h). We then found that LZTR1 expression was downregulated and negatively correlated with LL22NC03-N14H11.1 expression in HCC tissues (Fig. 4i, j). The low expression of LZTR1 in HCC cell lines vs. normal cell line was also verified (Fig. 4k). Collectively, LL22NC03-N14H11.1 inhibited LZTR1-mediated ubiquitination of H-RAS (G12V) and activated MAPK pathway to induce p-DRP1 (S616).

### LL22NC03-N14H11.1 transcriptionally repressed LZTR1 by recruiting c-Myb

**Fig. 5:**
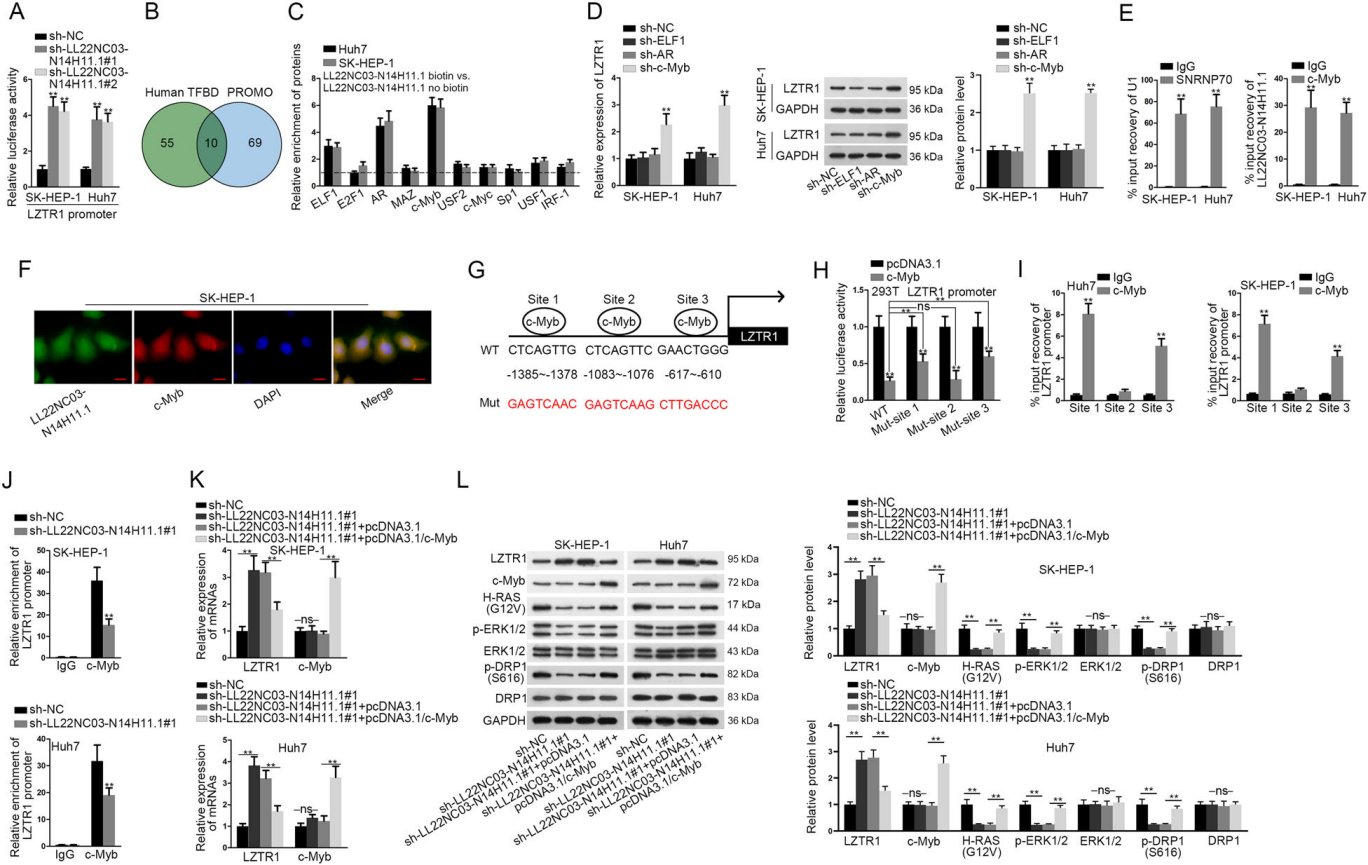
LL22NC03-N14H11.1 transcriptionally inhibited ZLTR1 through recruiting c-Myb. **a** Luciferase activity of LZTR1 promoter reporter in HCC cells under LL22NC03-N14H11.1 silence. **b** Ten transcription factors potentially targeting LZTR1 promoter was predicted by human TFDB (*P* < 0.001) and PROMO (*P* < 0.05). **c** The enrichment of ten predicted TFs in the pulldown of LL22NC03-N14H11.1 biotin vs. LL22NC03-N14H11.1 no biotin in HCC cells was quantified. **d** RT-qPCR assessed the expression of LZTR1 at mRNA and protein levels in HCC cells under the silence of ELF1, AR, and c-Myb, **e** RT-qPCR analysis of LL22NC03-N14H11.1 enrichment in precipitates of c-Myb after RIP assay. Interaction between U1 and SNRNP70 served as the positive control of this assay. **f** FISH staining of LL22NC03-N14H11.1 and IF analysis of c-Myb showed their co-localization in the nucleus of SK-HEP-1 cells. Scale bar: 25 μm. **g** Three predicted c-Myb sites on LZTR1 promoter. **h** Luciferase activity of wild-type (WT) LZTR1 promoter reporter or with site 1/2/3 mutated, respectively, in 293T cells under c-Myb overexpression. **i** RT-qPCR analysis of LZTR1 promoter with predicted c-Myb site 1, 2, or 3 in the RIP of c-Myb in HCC cells. **j** Enrichment of LZTR1 promoter in the ChIP complexes of c-Myb under LL22NC03-N14H11.1 silence vs. control. **k** HCC cells were transfected with sh-NC, sh-LL22NC03-N14H11.1#1, sh-LL22NC03-N14H11.1#1 + pcDNA3.1, or sh-LL22NC03-N14H11.1#1 + pcDNA3.1/c-Myb, respectively. RT-qPCR data of LZTR1 and c-Myb levels in HCC cells of each group. **l** Western blot results of LZTR1, c-Myb. H-RAS (G12V), p-ERK1/2, total ERK1/2, p-DRP1 (S616) and total DRP1 in HCC cells of each group. **P < 0.01; n.s. no significance.

Subsequently, we probed the mechanism whereby LL22NC03-N14H11.1 suppressed LZTR1 expression. We found through luciferase reporter assay that the transcription activity of LZTR1 was induced under LL22NC03-N14H11.1 depletion in HCC cells (Fig. 5a). LncRNAs are reported to interact with certain transcription factors to regulate target genes^40,41^. Since LL22NC03-N14H11.1 was identified to be mainly located in the nucleus of HCC cells, we deduced that LL22NC03-N14H11.1 could regulate the transcription of LZTR1 through affecting the function of certain transcription factors. We identified 65 TFs potentially targeting LZTR1 promoter from Human TFBD (http://211.67.31.242/HumanTFDB) (*P* < 0.001) and 69 TFs from PROMO (http://alggen.lsi.upc.es/cgi-bin/promo_v3/promo/promoinit.cgi?dirDB=TF_8.3) (*P* < 0.05). Intersection of the prediction results of two bioinformatics tools revealed 10 TFs targeting LZTR1 promoter (Fig. 5b). Pull-down analysis depicted that among 10 above-mentioned TFs, ELF1, AR, and c-Myb were significantly enriched in the pull-down compounds of the LL22NC03-N14H11.1 biotin group compared with the LL22NC03-N14H11.1 no biotin group (Fig. 5c). Furthermore, we observed an interesting phenomenon that the expression of LZTR1 was only induced in HCC cells after silencing c-Myb rather than other two TFs (Fig. 5d). c-Myb is a known TF that could either activate or suppress the transcription of target genes, and is involved in tumor-related activities, such as proliferation, stemness, and metastasis^42,43^. Recently, nuclear lncRNAs have been increasingly elucidated to recruit certain protein and serve as a scaffold to promote protein–protein or protein–RNA interactions^44^. Hence, we speculated that LL22NC03-N14H11.1 repressed LZTR1 transcription through recruiting c-Myb. RIP analysis confirmed the interaction between LL22NC03-N14H11.1 with c-Myb in HCC cells (Fig. 5e). FISH and IF staining revealed the co-localization of LL22NC03-N14H11.1 and c-Myb in the nucleus of SK-HEP-1 cells (Fig. 5f). We identified three putative c-Myb sites on LZTR1 through PROMO tool (with the lowest dissimilarity) as shown in Fig. 5g. Luciferase activity of wild-type LZTR1 promoter was reduced by c-Myb overexpression and mutating site 1 or 3 partially reversed such results. However, mutating site 2 could not restore the luciferase activity of LZTR1 promoter that was inhibited by c-Myb overexpression (Fig. 5h). ChIP analysis depicted that LZTR1 promoter fragments containing site 1 and 3, rather than site 2, could be immunoprecipitated by anti-c-Myb (Fig. 5i). Moreover, knockdown of LL22NC03-N14H11.1 reduced the enrichment of LZTR1 promoter in c-Myb precipitates (Fig. 5j). In contrast, the occupancy of c-Myb on LZTR1 promoter was obviously fortified by upregulated LL22NC03-N14H11.1 (Supplementary Fig. S2E). These data indicated that LL22NC03-N14H11.1 contributed to the binding of c-Myb to LZTR1 promoter. Then, we found that the level of LZTR1 induced by LL22NC03-N14H11.1 silence was reversed by c-Myb overexpression, but LL22NC03-N14H11.1 silence failed to influence c-Myb expression at the mRNA and protein levels (Fig. 5k, l). Also, levels of H-RAS (G12V), p-ERK1/2, and p-DRP1 (S616) reduced by sh-LL22NC03-N14H11.1#1 were recovered by overexpression of c-Myb (Fig. 5l). Altogether, LL22NC03-N14H11.1 transcriptionally repressed LZTR1 through recruiting c-Myb to LZTR1 promoter, resulting in activated H-RAS/MAPK pathway.

### LL22NC03-N14H11.1 promoted mitochondrial fission and HCC progression through LZTR1/H-RAS/MAPK pathway

**Fig. 6:**
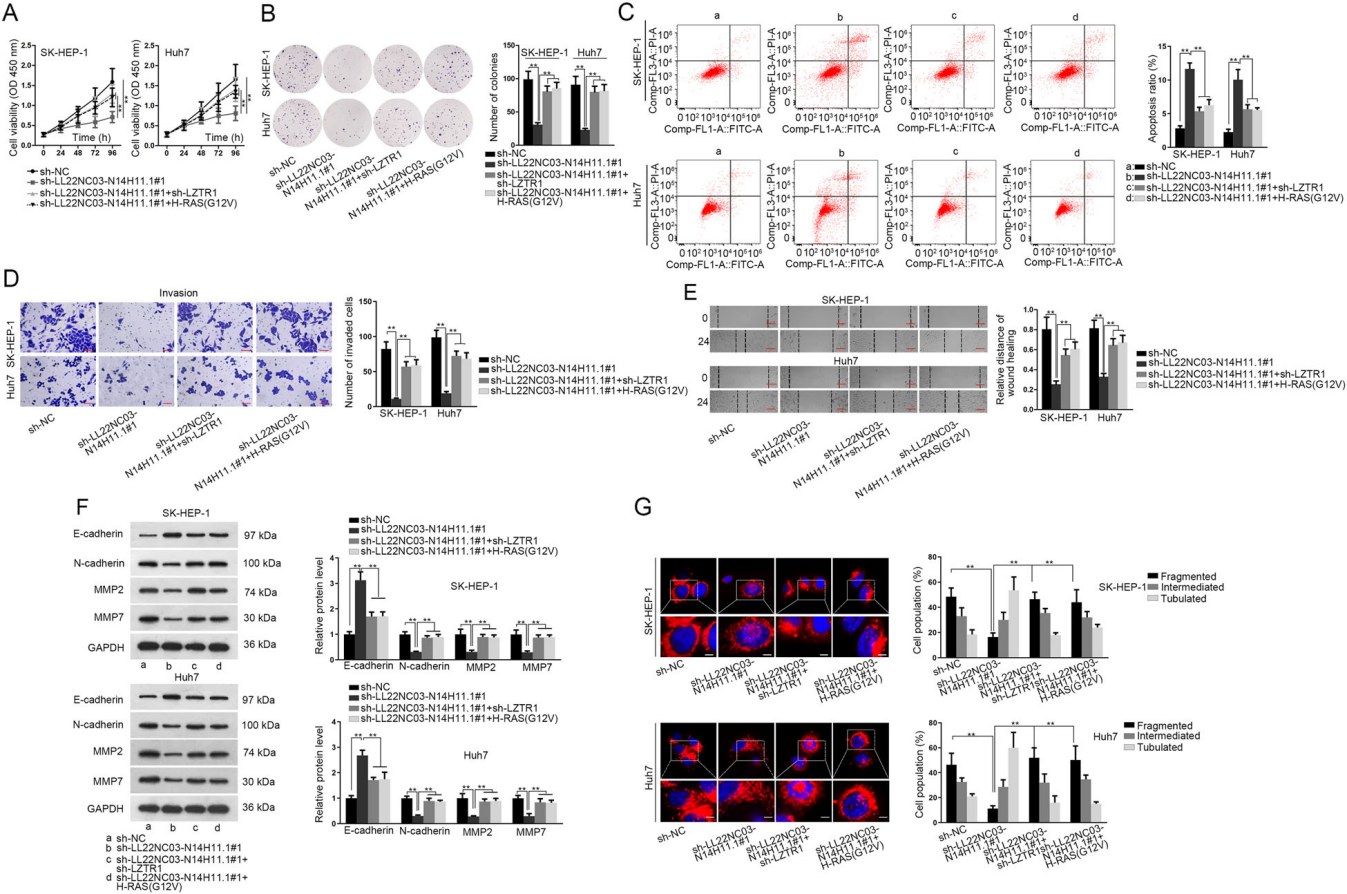
LL22NC03-N14H11.1 regulated HCC progression and mitochondrial fission through LZTR1/H-RAS (G12V). **a**–**e** HCC cells were transfected with sh-NC (**a**), sh-LL22NC03-N14H11.1#1 (**b**), sh-LL22NC03-N14H11.1#1 + sh-LZTR1 (**c**), or sh-LL22NC03-N14H11.1#1 + H-RAS (G12V) (**d**), respectively. Proliferation, apoptosis, invasion, and migration of HCC cells were evaluated by CCK-8, colony formation, transwell invasion, and wound healing assays, respectively. **f** Western blot analysis of E-cadherin, N-cadherin, MMP2, and MMP7 in HCC cells of each group. **g** MitoTracker Red staining and quantification of mitochondrial fission in HCC cells of each group. Scale bar: 3 μm. **P < 0.01.

To detect whether LL22NC03-N14H11.1 regulated HCC progression and mitochondrial fission through LZTR1/H-RAS/MAPK pathway, we designed rescue experiments. As a result, either LZTR1 knockdown or H-RAS (G12V) overexpression rescued proliferation of HCC cells with LL22NC03-N14H11.1 silence (Fig. 6a, b). Apoptosis of HCC cells increased by LL22NC03-N14H11.1 knockdown was reversed by LZTR1 silence or H-RAS (G12V) overexpression (Fig. 6c). Inhibitory effect of LL22NC03-N14H11.1 depletion on HCC cell invasion and migration was abrogated by silencing LZTR1 or overexpressing H-RAS (G12V) (Fig. 6d, e). LZTR1 depletion or H-RAS (G12V) overexpression reversed the increase of E-cadherin and decrease of N-cadherin, MMP2, and MMP7 in LL22NC03-N14H11.1-silenced HCC cells (Fig. 6f). The attenuated mitochondrial fission under LL22NC03-N14H11.1 silence in HCC cells was restored by the knockdown of LZTR1 or overexpression of H-RAS (G12V) (Fig. 6g). Altogether, LL22NC03-N14H11.1 aggravated malignant phenotypes in HCC through targeting LZTR1/H-RAS/MAPK pathway.

### LL22NC03-N14H11.1 facilitated tumor growth and metastasis of HCC in vivo

**Fig. 7:**
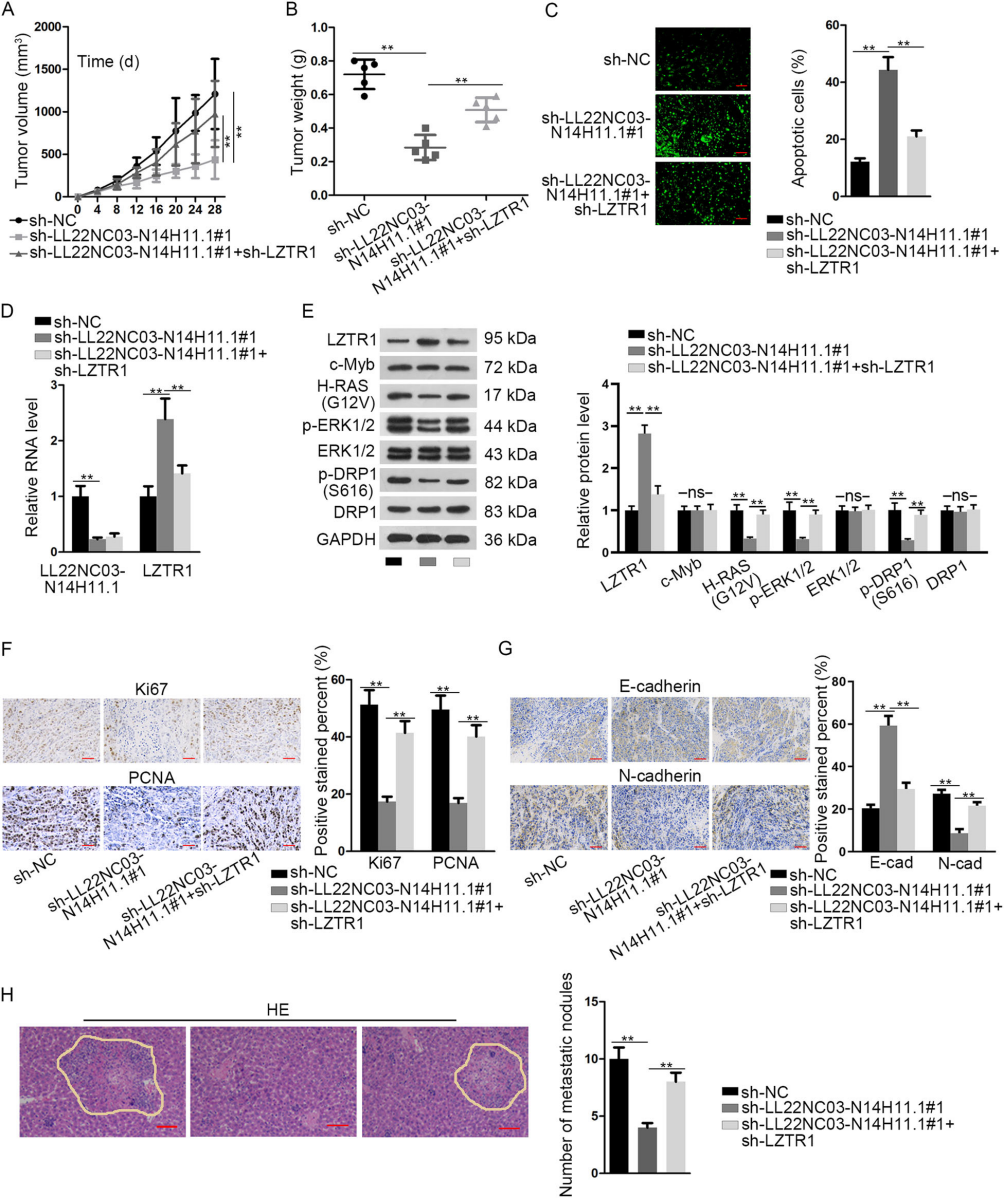
LL22NC03-N14H11.1 drove tumorigenesis and metastasis in HCC through LZTR1 in vivo. SK-HEP-1 cells were, respectively, transfected with sh-NC, sh-LL22NC03-N14H11.1#1, or sh-LL22NC03-N14H11.1#1 + sh-LZTR1 and injected subcutaneously or from tail vain to monitor tumorigenesis and metastasis of HCC. **a** Volumes of xenografts in mice of each group was evaluated every 4 days after subcutaneous injection and the growth curve was outlined. **b** Twenty-eight days after injection, tumors from each group were resected and weighed. **c** TUNEL staining was used to evaluate the apoptosis in tumors of each group. **d** RT-qPCR analysis of LL22NC03-N14H11.1 and LZTR1 in tumors of each group. **e** Western blot was implemented to detect the levels of LZTR1, c-Myb, H-RAS (G12V), p-ERK1/2, total ERK1/2, p-DRP1 (S616), and total DRP1 in tumors of each group. **f**, **g** IHC staining and quantification of Ki67, PCNA, N-cadherin, and E-cadherin in tumors of each group. **h** Metastatic nodules in tumors of each group was stained by HE and quantified. Scale bar: 100 μm. **P < 0.01; n.s. no significance.

Finally, in vivo assays were conducted to confirm the role of LL22NC03-N14H11.1 in HCC. SK-HEP-1 cells were transfected with sh-NC, sh-LL22NC03-N14H11.1#1, or sh-LL22NC03-N14H11.1#1 + sh-LZTR1, respectively, and subcutaneously injected into nude mice to establish xenografts. Consequently, LL22NC03-N14H11.1 knockdown retarded tumor growth of HCC in vivo, but co-transfection of sh-LZTR1 abrogated such effect (Fig. 7a). LL22NC03-N14H11.1 depletion reduced tumor weight, and co-transfection of sh-LZTR1 restored such reduction (Fig. 7b). The apoptosis of HCC cells induced by sh-LL22NC03-N14H11.1#1 in vivo was impaired by LZTR1 silence (Fig. 7c). RT-qPCR data confirmed that knockdown of LL22NC03-N14H11.1 reduced LL22NC03-N14H11.1 level and induced LZTR1 level, and co-transfection of sh-LZTR1 impaired the induction of LZTR1 level, but had no influence on LL22NC03-N14H11.1 expression (Fig. 7d). Western blot analysis verified that LL22NC03-N14H11.1 knockdown induced LZTR1, but decreased H-RAS (G12V), p-ERK1/2, and p-DRP1 (S616) in vivo, and such results were reversed by LZTR1 silence, with c-Myb, total ERK1/2, and total DRP1 levels unchanged all the way (Fig. 7e). IHC staining exhibited that the positivity of proliferation index (Ki67 and PCNA) and mesenchymal marker (N-cadherin) was reduced, and epithelial marker (E-cadherin) was induced in xenografts with LL22NC03-N14H11.1 silence, while knockdown of LZTR1 reversed these results (Fig. 7f–g). HE staining revealed that the number of in vivo metastatic nodules was lessened by LL22NC03-N14H11.1 knockdown, but recovered by the silence of LZTR1 (Fig. 7h). In conclusion, it was suggested that LL22NC03-N14H11.1 facilitated tumor growth and metastasis of HCC through LZTR1 in vivo.

## Discussion

Studies have provided convincing evidences that hepatocarcinogenesis involves dysregulation of lncRNAs. Large amounts of lncRNAs are identified to possess prognostic and diagnostic values in HCC^8,9^. Therefore, identifying novel lncRNAs in HCC and finding out their modulatory mechanism can benefit the progress of treatment efficacy in HCC and improve the survival of HCC patients.

This study analyzed the dysregulated lncRNAs in HCC through two bioinformatics tools (circlncRNAnet and GEPIA) and found that LL22NC03-N14H11.1 was a new lncRNA highly expressed in HCC samples and had great prognostic significance for HCC patients. We also confirmed that LL22NC03-N14H11.1 level was elevated in HCC tissues and cell lines. Besides, we found that LINC00152 was also highly expressed in HCC samples. A number of former studies have elucidated that LINC00152 served as an oncogene in HCC^36–38^, but the role of LL22NC03-N14H11.1 in HCC has never been investigated. Therefore, we focused on the exploration of LL22NC03-N14H11.1 in HCC. Functionally, we discovered that LL22NC03-N14H11.1 accelerated proliferation, migration, invasion, and EMT in HCC cells. These findings indicated that targeting LL22NC03-N14H11.1 in HCC might be a promising approach to relieve hepatocarcinogenesis.

Mitochondrial fission has been demonstrated as an important activity related to cell survival and metastasis in tumors. Some works stated that increased mitochondrial fission contributes to drug-induced apoptosis and enhance chemo-sensitivity in cancer cells^45^, whereas others argued that inhibiting mitochondrial fission can retard cell proliferation and metastasis in several cancer types, such as colon cancer, lung cancer, and breast cancer^14–18^. A recent study observed that mitochondrial fission was facilitated in HCC samples compared to adjacent normal samples, and that DRP1 inhibition could inhibit cell survival and autophagy in HCC^19^, indicating that excessive mitochondrial fission in HCC was oncogenic. In this study, we found that LL22NC03-N14H11.1 silence prevented mitochondrial fission, same as the effect of Midivi-1 (DRP1 inhibitor). Also, we validated that overexpressing DRP1 could rescue the inhibitory effect of LL22NC03-N14H11.1 silence on HCC progression, indicating that LL22NC03-N14H11.1 regulated HCC through facilitating DRP1-regulated mitochondrial fission.

DRP1 is a well-known member of dynamin-related GTPases, which were referred to as primary regulators of mitochondrial dynamics^13,20^. It has been well established that phosphorylation of DRP1 at S616 led to DRP1 activation and facilitated mitochondrial fission^21^. Herein, we found that LL22NC03-N14H11.1 contributed to DRP1 phosphorylation at S616, with no impact on the level of total DRP1. Previous works have identified several regulators of p-DRP1 (S616), such as CDK1, DPI1, and ERK1/2^22–24^. Herein, we discovered that among the aforementioned regulators, only ERK1/2 phosphorylation was positively regulated by LL22NC03-N14H11.1 in HCC cells. ERK1/2 was the key regulators in ERK/MAPK pathway. It is widely known that MAPK pathway is a key signaling related to the survival, apoptosis, and metastasis in tumor cells^46,47^. Recent studies revealed that H-RAS (G12V) activated MAPK pathway so that ERK1/2 was phosphorylated and induced p-DRP1 (S616)^25^. Importantly, such mechanism has been proved to accelerate mitochondrial fission and tumor growth in pancreatic cancer^26^. Hence, it was reasonable to deduce that LL22NC03-N14H11.1 regulated MAPK pathway to induce mitochondrial fission in HCC.

Expectedly, we firstly found that LL22NC03-N14H11.1 knockdown reduced H-RAS (G12V) level in HCC cells. We further suggested that LL22NC03-N14H11.1 regulated H-RAS (G12V) protein stability. Several studies have pointed out that LZTR1 was a conserved regulator of the ubiquitination of RAS family. LZTR1 was suggested to possess tumor-suppressing function in cancers and be related to cell apoptosis^32–34^. A study elucidated that LZTR1 ubiquitinated K-RAS, M-RAS, N-RAS, H-RAS, and their mutant types, leading to the inactivation of MAPK pathway^35^. Therefore, it was reasonable to suggest that LZTR1 exerted tumor-suppressive effect through suppressing MAPK pathway, and that LZTR1 might mediate the regulation of LL22NC03-N14H11.1 on H-RAS (G12V) in HCC. This study firstly revealed that LL22NC03-N14H11.1 reduced LZTR1 expression to inhibit the LZTR1-mediated ubiquitination of H-RAS (G12V).

Furthermore, we uncovered that LL22NC03-N14H11.1 repressed LZTR1 expression at the transcriptional level. We identified that LL22NC03-N14H11.1 was mainly located in the nucleus of HCC cells. The mechanisms whereby lncRNAs regulate target genes are different depending on their cellular localization^10,11^. In the nucleus, lncRNAs can recruit certain transcription factors to affect the transcription of target genes^40,41^. For instance, lncRNA REG1CP could tether FANCJ to REG3A promoter to induce REG3A activation by interacting with FANCJ in colorectal cancer^48^. Also, a latest research discovered that lncRNA Oplr16 could activate Oct4 via inducing DNA demethylation by recruiting TET2^49^. Herein, we identified that c-Myb potentially targeted LZTR1 promoter and interacted with LL22NC03-N14H11.1. As a transcription factor, c-Myb is demonstrated to either suppress or activate gene transcription^42,43^, and it has been proved to contribute to hepatocarcinogenesis and metastasis in HCC^50^. In this study, we validated that LL22NC03-N14H11.1 recruited c-Myb to LZTR1 promoter so as to repress LZTR1 transcription and activate H-RAS/MAPK pathway, suggesting LL22NC03-N14H11.1 as a scaffold in HCC as many other lncRNAs do^44,51^. Besides, it has been revealed that c-Myb could repress transcription by recruiting certain transcription repressor or competing with other transcription activators for target promoters^43^. However, the precise mechanism whereby c-Myb inhibited LZTR1 transcription remains yet to be further investigated in the future. Finally, we validated that LL22NC03-N14H11.1 promoted HCC tumorigenesis and metastasis in vivo.

## Conclusions

**Fig. 8:**
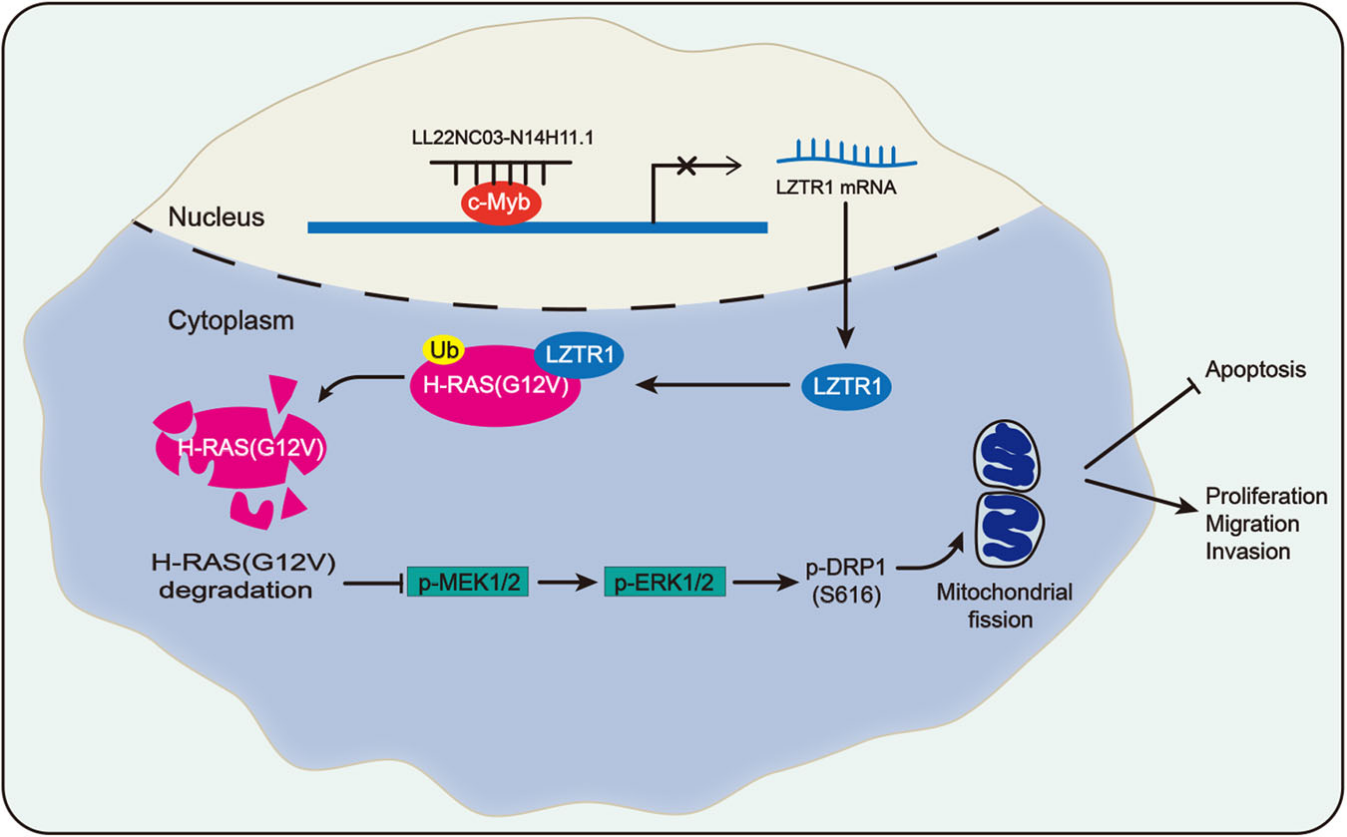
Graphical presentation of LL22NC03-N14H11.1/c-Myb/LTZR1/H-RAS/MAPK/DRP1 axis in HCC. LL22NC03-N14H11.1 interacted with c-Myb to transcriptionally repress LZTR1, so as to decrease H-RAS (G12V) ubiquitination, activate MAPK pathway, and induce p-DRP1 (S616), resulting in induced mitochondrial fission and accelerated HCC progression.

This study firstly revealed a novel lncRNA LL22NC03-N14H11.1 as an oncogene in HCC. Functionally, LL22NC03-N14H11.1 promoted proliferation, invasion, migration, and prevented apoptosis in vitro and drove tumorigenesis and metastasis in vivo. Besides, LL22NC03-N14H11.1 promoted p-DRP1 (S616) to facilitate mitochondrial fission. Mechanistically, LL22NC03-N14H11.1 interacted with c-Myb to transcriptionally repress LZTR1, so as to decrease H-RAS (G12V) ubiquitination, activate MAPK pathway, and induce p-DRP1 (S616) (Fig. 8). These findings indicated LL22NC03-N14H11.1 as a new target in HCC and might provide new thoughts for the lncRNA-targeted molecular therapy in HCC.

## Supplementary information

Figure S1

Figure S2

supplementary figure legends
