# Supplementary figures and images for: LncRNA LL22NC03-N14H11.1 promoted hepatocellular carcinoma progression through activating MAPK pathway to induce mitochondrial fission
Source: Cell Death Dis. 2020 Oct 7;11(10):832. doi: 10.1038/s41419-020-2584-z (PMC7542152; doi:10.1038/s41419-020-2584-z)

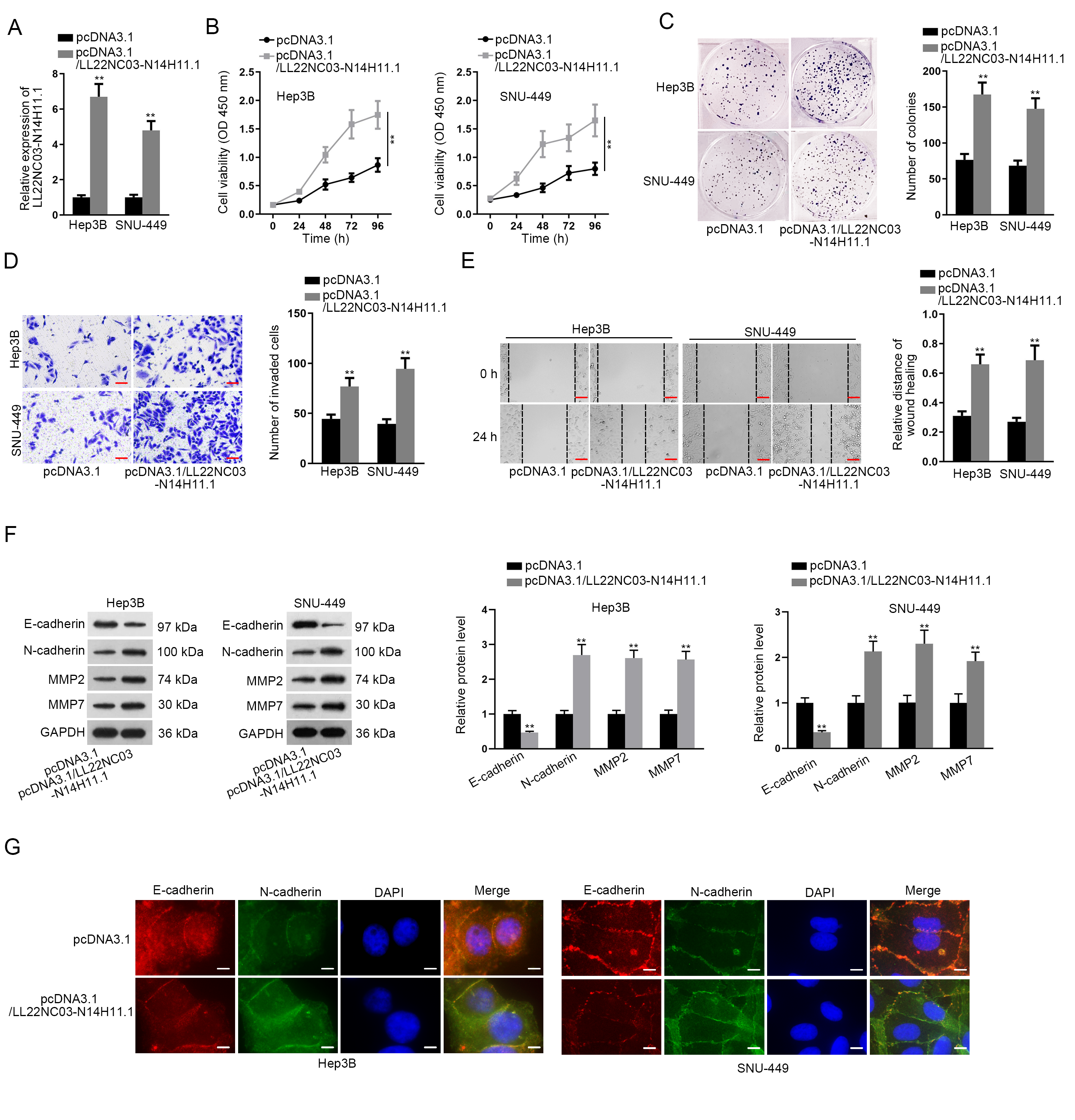

Supplement: Supplementary file 1 — Figure S1 [file 41419_2020_2584_MOESM1_ESM.tif]

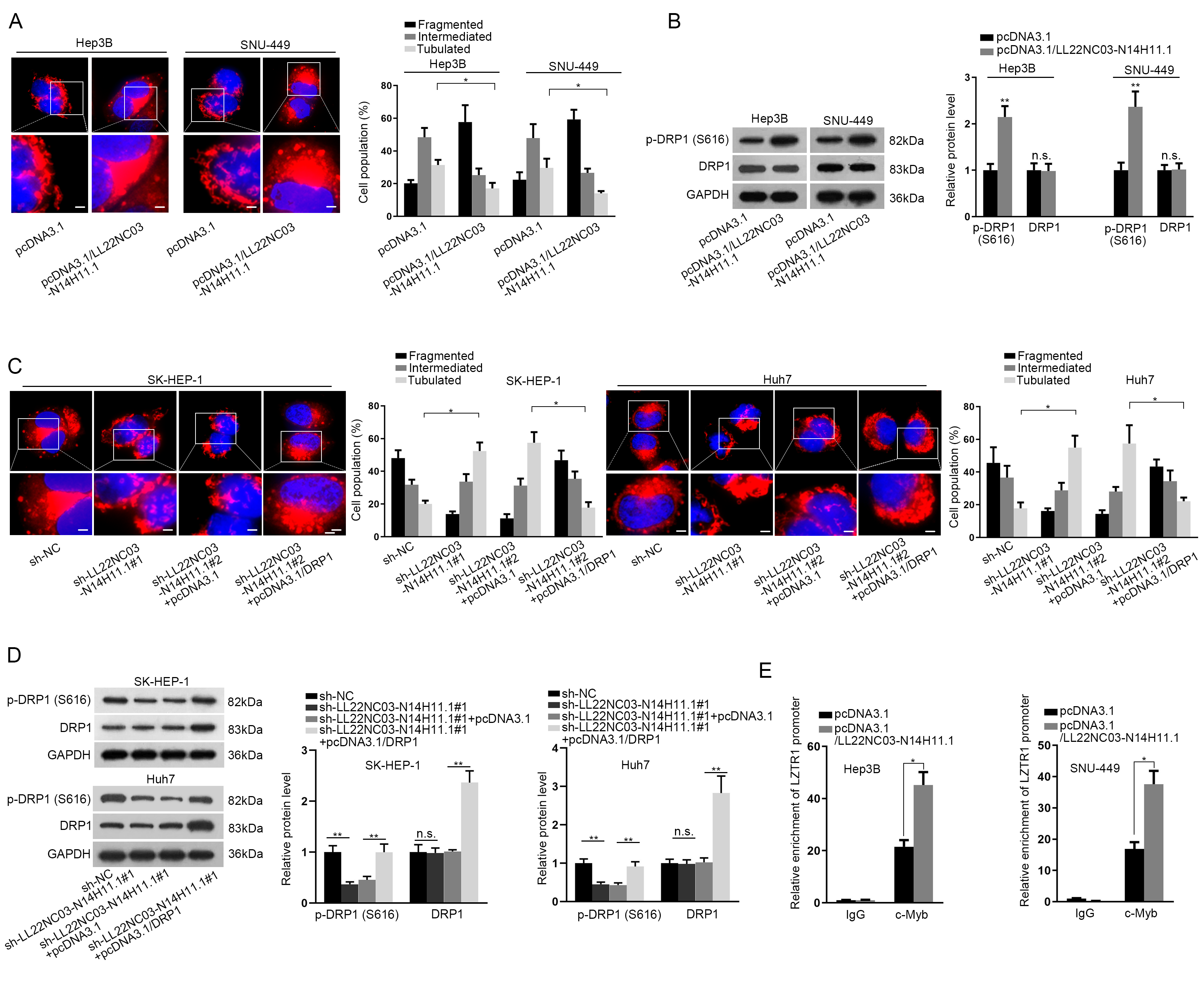

Supplement: Supplementary file 2 — Figure S2 [file 41419_2020_2584_MOESM2_ESM.tif]
