# Supplementary material for: LncRNA LL22NC03-N14H11.1 promoted hepatocellular carcinoma progression through activating MAPK pathway to induce mitochondrial fission
Source: Cell Death Dis. 2020 Oct 7;11(10):832. doi: 10.1038/s41419-020-2584-z (PMC7542152; doi:10.1038/s41419-020-2584-z)
Supplement: Supplementary file 3 — supplementary figure legends [file 41419_2020_2584_MOESM3_ESM.docx]

**Figure S1 (A)** RT-qPCR data of LL22NC03-N14H11.1 level in Hep3B and SNU-449 cells transfected with pcDNA3.1 or pcDNA3.1/LL22NC03-N14H11.1. **(B-C)** The impact of LL22NC03-N14H11.1 overexpression on cell viability and proliferation was evaluated by CCK8 and colony formation assays. **(D-E)** The influence of elevated LL22NC03-N14H11.1 on cell invasion and migration was determined via transwell assay and wound healing assay, respectively. **(F)** Western blot results of E-cadherin, N-cadherin, MMP2, and MMP7 in HCC cells with LL22NC03-N14H11.1 upregulation. **(G)** IF staining of E-cadherin and N-cadherin in two HCC cells with or without LL22NC03-N14H11.1 overexpression. Scale bar: 50μm. ^**^P < 0.01.

**Figure S2 (A)** Mitochondrial fission in Hep3B and SNU-449 cells transfected with pcDNA3.1 or pcDNA3.1/LL22NC03-N14H11.1 was monitored by MitoTracker Red staining. Scale bar: 3μm. **(B)** Western blot results of p-DRP1 (S616) and total DRP1 in above Hep3B and SNU-449 cells. **(C)** MitoTracker Red staining was performed to monitor mitochondrial fission in SK-HEP-1 and Huh7 cells transfected with sh-NC, sh-LL22NC03-N14H11.1#1, sh-LL22NC03-N14H11.1#2+pcDNA3.1 or sh-LL22NC03-N14H11.1#2+pcDNA3.1/DRP1. Scale bar: 3μm. **(D)** Western blot results of p-DRP1 (S616) and total DRP1 in SK-HEP-1 and Huh7 cells under above conditions. **(E)** The effect of LL22NC03-N14H11.1 upregulation on the binding of c-Myb to LZTR1 promoter was assessed via ChIP assay. ^**^P < 0.01.
